# Overcoming the Challenges of BeiDou Receiver Implementation

**DOI:** 10.3390/s141122082

**Published:** 2014-11-21

**Authors:** Mohammad Zahidul H. Bhuiyan, Stefan Söderholm, Sarang Thombre, Laura Ruotsalainen, Heidi Kuusniemi

**Affiliations:** Department of Navigation and Positioning, Finnish Geodetic Institute, 02430 Kirkkonummi, Finland; E-Mails: stefan.soderholm@fgi.fi (S.S.); sarang.thombre@fgi.fi (S.T.); laura.ruotsalainen@fgi.fi (L.R.); heidi.kuusniemi@fgi.fi (H.K.)

**Keywords:** BeiDou Navigation Satellite system, acquisition, tracking, software-defined receiver, multi-GNSS

## Abstract

Global Navigation Satellite System (GNSS)-based positioning is experiencing rapid changes. The existing GPS and the GLONASS systems are being modernized to better serve the current challenging applications under harsh signal conditions. These modernizations include increasing the number of transmission frequencies and changes to the signal components. In addition, the Chinese BeiDou Navigation Satellite system (BDS) and the European Galileo are currently under development for global operation. Therefore, in view of these new upcoming systems the research and development of GNSS receivers has been experiencing a new upsurge. In this article, the authors discuss the main functionalities of a GNSS receiver in view of BDS. While describing the main functionalities of a software-defined BeiDou receiver, the authors also highlight the similarities and differences between the signal characteristics of the BeiDou B1 open service signal and the legacy GPS L1 C/A signal, as in general they both exhibit similar characteristics. In addition, the authors implement a novel acquisition technique for long coherent integration in the presence of NH code modulation in BeiDou D1 signal. Furthermore, a simple phase-preserved coherent integration based acquisition scheme is implemented for BeiDou GEO satellite acquisition. Apart from the above BeiDou-specific implementations, a novel Carrier-to-Noise-density ratio estimation technique is also implemented in the software receiver, which does not necessarily require bit synchronization prior to estimation. Finally, the authors present a BeiDou-only position fix with the implemented software-defined BeiDou receiver considering all three satellite constellations from BDS. In addition, a true multi-GNSS position fix with GPS and BDS systems is also presented while comparing their performances for a static stand-alone code phase-based positioning.

## 1. Introduction

The Chinese BeiDou Navigation Satellite system (BDS) has a mixed space constellation that will have, when fully deployed, five Geostationary Earth Orbit (GEO) satellites, twenty-seven MEO satellites and three Inclined Geosynchronous Satellite Orbit (IGSO) satellites. The GEO satellites are operating in orbit at an altitude of 35,786 kilometers and positioned at 58.75°E, 80°E, 110.5°E, 140°E and 160°E, respectively. The MEO satellites are operating in orbit at an altitude of 21,528 km and an inclination of 55° to the equatorial plane. The IGSO satellites are operating in orbit at an altitude of 35,786 km and an inclination of 55° to the equatorial plane. These satellites broadcast navigation signals and messages within three frequency bands. The BDS has been in development for more than a decade, and it is estimated to be operational with global coverage at the latest in 2020 [1,2]. The BeiDou satellites transmit ranging signals based on Code Division Multiple Access (CDMA) principle, like GPS and Galileo. The mixed constellation structure of BeiDou results in better observation geometry for positioning and orbit determination compared to current GPS and GLONASS, and future Galileo, especially in China and neighboring regions. The BeiDou system has already started contributing to the multi-GNSS benefits where increased accuracy, availability and integrity are possible when utilizing interoperable GNSS [3].

The characteristics of BeiDou B1I (B1 In-phase) signal can be compared with GPS L1 signal in order to realize the similarities and differences between the two systems. Both the civilian signals from these two systems have similar characteristics in general, for example, the periods of their spreading codes are both 1 millisecond (ms) long, and the coordinate systems and the navigation message structures are almost the same with minor differences [4,5]. This eventually means that many algorithms that are implemented for the GPS receiver can be readily available to the BeiDou receiver without any major modification. But to improve the positioning performance, the modern GNSS signals, including BeiDou and the GPS L5, introduce a second layer of modulation between the navigation data and the PRN code chips, known as Neumann-Hoffman (NH) code modulation. This ultimately improves the data bit rate of the modern GNSS signals.

The legacy GPS L1 C/A signal has a data bit rate of 50 bits per second (bps), which means that 1 bit data lasts for 20 ms (*i.e.*, the PRN code cycle repeats 20 times for each data bit). The data bit rate of BeiDou D2 signal is 500 bps, which means that 1 bit of data lasts for only 2 ms (*i.e.*, two spreading code cycles). The data bit rate of BeiDou D1 signals is originally 50 bps, but after modulation by NH code, the data bit rate becomes 1 kbps, so compared to the GPS signal, the data bit rate of the BeiDou signal increases significantly. Particularly, the NH code modulated D1 signal has 1 kbps data bit rate which makes data bit transition possible within the data bit boundary. The use of NH code and the resultant increase in the data bit rate has pros and cons. On the positive side, the NH code can boost the ability of anti-narrowband interference and improve the cross-correlation property of satellite signals and the bit synchronization [6]; whereas on the negative side, the existence of NH code makes the acquisition and tracking of the modernized GNSS signals more challenging [7–12]. The acquisition and tracking challenges in BDS will be discussed in more detail in Sections 2.1 and 2.2, respectively.

The use of a software-defined GNSS receiver is highly appreciated for its flexibility, re-configurability and diversity. These unique characteristics of a software-defined receiver make it possible to develop and then to validate new algorithms for optimizing the receiver performance at a low cost [13]. A number of software-defined receivers have already been developed for GNSS signal reception and processing [13–17]. Most of these receivers are capable of processing GPS, GLONASS and Galileo signals. Recently, a PC-based BeiDou software receiver was introduced in [18] albeit with limited algorithmic details on how to acquire, track and process a NH code modulated BeiDou signal. Therefore, in this paper, the authors discuss the implementation issues of a software-defined BeiDou receiver considering the challenges introduced by the existence of NH code modulation in case of BeiDou D1 signal and the higher data rate in case of BeiDou D2 signal.

The rest of this paper is organized as follows: Section 2 discusses the main functionalities of a software-defined GNSS receiver in view of BDS. In Section 3, a live data collection scenario, front-end configurations and positioning results are presented and discussed. Finally, conclusions and future work are discussed in Section 4.

## 2. Software-Defined GNSS Receiver

A GNSS software-defined receiver consists of three major components: RF front-end unit, a signal processing unit, and a navigation processing unit. The RF front-end module is responsible for signal amplification, noise filtering, down-conversion, automatic gain control, and analogue-to-digital conversion. The front-end module converts the received analog data to digital Intermediate Frequency (IF) data at a rate which is several times more than the code chipping rate. A 26 MHz sampling frequency is used to generate the raw digitized IF samples in all the experimented cases of this work.

The digital IF data are then processed by a signal processing unit whose main responsibilities include signal acquisition, code and carrier tracking and data demodulation. The demodulated data and the resulting pseudorange measurements are then utilized by a navigation processing unit in order to offer a Position, Velocity and Timing (PVT) solution, along with some other relevant information. The software-defined receiver differs from a conventional receiver in the sense that the functions of the processing and navigation units, including correlation/tracking and navigation tasks, are delivered by software, leading to a more flexible design with potential savings in cost and power.

**Figure 1.**
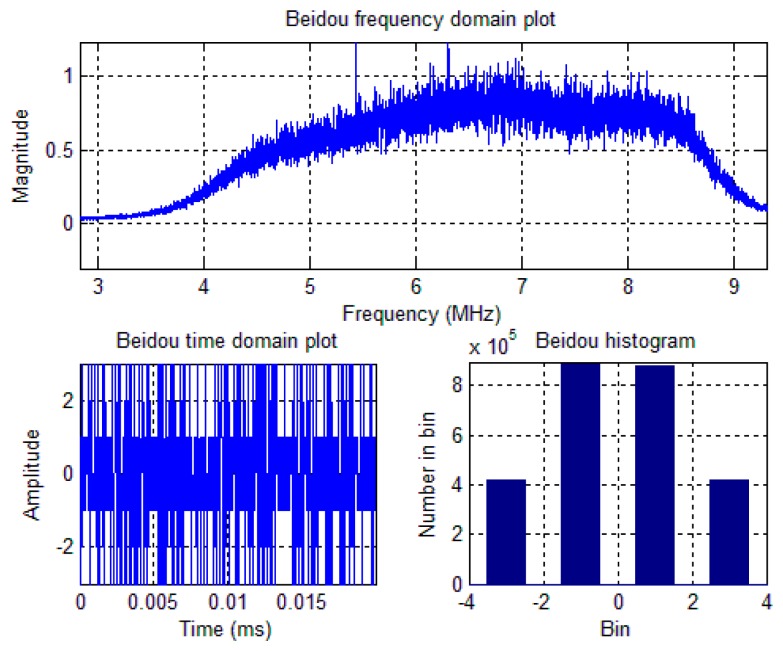
BeiDou signal spectrum (**Top**); time-domain plot (**Bottom-left**); and bin distribution (**Bottom-right**) of the digitized IF samples.

A software-defined GNSS receiver platform, named as FGI-GSRx (Version 2), has been developed in Finnish Geodetic Institute for the analysis and validation of novel algorithms for an optimized GNSS navigation performance. The basic version of FGI-GSRx is based on an open-source software receiver platform [13], and it has been adapted to be BeiDou-compatible with a dual-frequency front-end from Nottingham Scientific Limited (NSL) [18]. The NSL front-end, ‘stereo v2’ is used to capture the BeiDou data. The stereo front-end configuration is presented in Table 1. The BeiDou B1I signal spectrum, time-domain plot and bin distribution of the digitized IF samples are shown in Figure 1.

### 2.1. BeiDou Signal Acquisition

The main task at the signal acquisition stage is to determine which of the satellites are visible to the user and then, to coarsely estimate the carrier Doppler and the code phase of those visible satellites. Generally speaking, signal acquisition is a three dimensional search, where the satellite PRN number, the carrier Doppler and the code phase are coarsely estimated. An FFT-based signal acquisition technique is implemented in the software receiver.

#### 2.1.1. BeiDou IGSO and MEO Satellites Acquisition

The traditional coherent acquisition techniques used for GPS L1 C/A signal acquisition cannot directly be applied to acquire BeiDou D1 signal transmitted from IGSO and MEO satellites due to the presence of NH code modulation. The presence of NH code modulation on top of navigation data bits increases the final data bit rate from 50 bps to 1 kHz. Therefore, the sign changes for BeiDou D1 signal occur more frequently than that of a GPS L1 C/A signal due to the presence of NH code. This eventually means that an acquisition scheme for BeiDou D1 signal with more than 1 ms coherent integration period may appear dangerous, if sign information is not consistently preserved. In view of this sign transition problem, a novel acquisition technique is implemented for BeiDou D1 signal that preserves the total useful signal energy in the presence of a sign transition, and hence, makes a correct acquisition decision on the presence of the satellite, and its carrier Doppler and the corresponding code phase. The working principle of the novel BeiDou D1 acquisition technique, first introduced in [9], is also depicted in the following.

**Figure 2.**
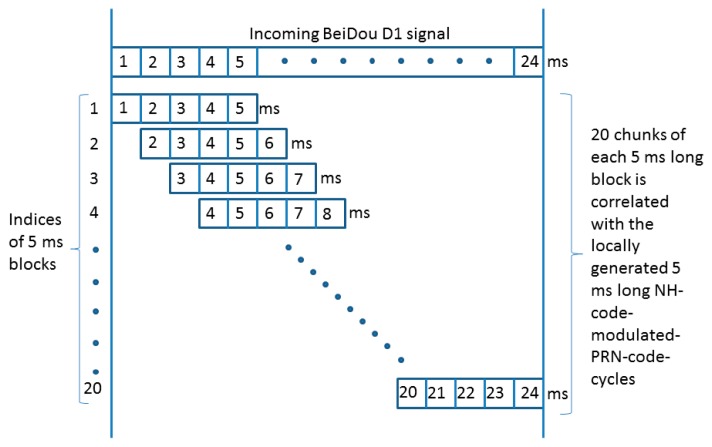
24 ms long incoming BeiDou signal is structured into 20 chunks of 5 ms long block with an index increment of 1.

**Figure 3.**
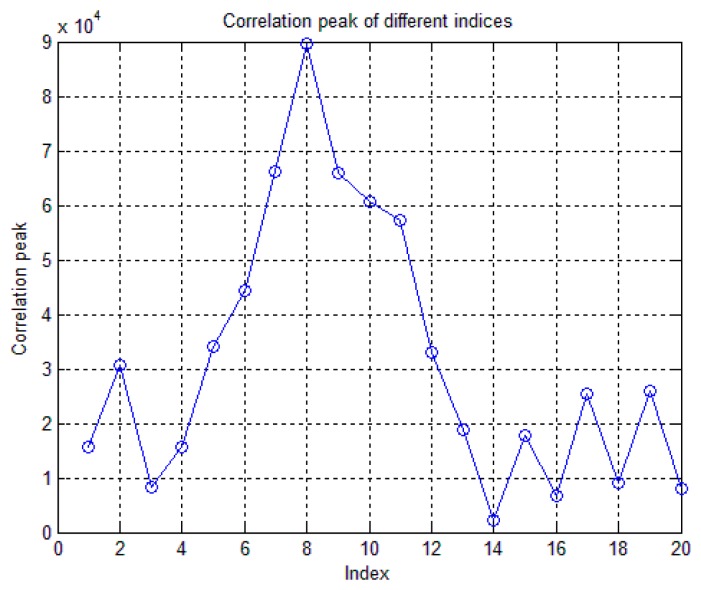
Acquisition correlation peaks for different NH indices.

Step 1:For a coherent integration period of *T_coh_* ms, a (*T_coh_* × 1000 =) *X_bit_* number of NH code bits is selected first. For example, for a coherent integration period of 5 ms, the first 5 bits of NH code, *i.e.*, [−1 −1 −1 −1 −1] can be selected. Also, a long incoming BeiDou signal of (*T_coh_* + 19) ms is required to carry out the FFT-based acquisition. In case of *T_coh_* = 5 ms, the acquisition metric will be consisted of 24 ms long incoming signal.Step 2:The frequency resolution is chosen such that the frequency bin size is less than (2/3) × *T_coh_*, where *T_coh_* is the coherent integration time. In case of a 5 ms integration time, the frequency bin size should be less than or equal to 133.33 Hz.Step 3:The chosen *X_bit_* long NH code sequence is then multiplied with the locally generated BeiDou PRN codes in order to form an *X_bit_* long NH-code-modulated-PRN-code-cycle.Step 4:An FFT-based correlation is then performed on each *T_coh_* ms blocks of incoming BeiDou signal with the locally generated *X_bit_* long NH-code-modulated-PRN-code-cycles (*i.e.*, the output of Step 3 with an incoming signal index increment of 1. An example on how the incoming BeiDou D1 signal is structured for acquisition is shown in Figure 2 below for a coherent integration period of *T_coh_* = 5 ms.Step 5:As the NH code length is 20 bits, there are altogether 20 chunks of correlation matrices with all possible code delay and carrier Doppler combinations for a specific BeiDou satellite. The winning index is the one which has the maximum correlation peak as shown in Figure 3. Therefore, this winning NH index can be used for detecting the presence of the satellite, along with the estimation of the carrier Doppler and the code phase via a pre-detection threshold computed against a certain probability of false alarm.

#### 2.1.2. BeiDou GEO Satellites Acquisition

In case of BeiDou GEO satellites which transmit D2 navigation signal, there is no NH code modulation on top of the data bits. But, D2 signal has a data bit rate of 500 bps, meaning that each data bit will last for 2 ms. Therefore, the bit transition in case of BeiDou D2 signal may occur after every 2 ms unlike the GPS L1 C/A signal, where the bit transition may occur only after 20 ms. In view of this much frequent sign transition occurrences, a similar strategy like D1 signal acquisition technique is implemented for BeiDou D2 signal. The implementation strategy for D2 signal acquisition is described in the following.

Step 1:The chosen number of coherent integration summation should be even (*i.e.*, multiple of 2 due to the bit interval duration of D2 signal).Step 2:For a coherent integration period of *T_coh_* ms (which is chosen as even), a (((*T_coh_*)/2) × 1000 =) *X_bit_* number of data bits is selected first. For example, for a coherent integration period of 4 ms, the number of data bits, *X_bit_* = 2. In case of *X_bit_* = 2, all possible combinations for incoming data bits will be 2*^X^_bit_* = 4. Therefore, there will be four possibilities for incoming received data bits, which are: [+1 +1], [+1 −1], [−1 +1], and [+1 +1].Step 3:The frequency resolution is chosen such that the frequency bin size is less than (2/3) × *T_coh_*, where *T_coh_* is the coherent integration time. In case of a 4 ms integration time, the frequency bin size should be less than or equal to 166.67 Hz.Step 4:Each of the 2*^X^_bit_* data-bit set is then multiplied with the locally generated BeiDou PRN codes in order to form a *T_coh_* ms long data-modulated-PRN-code-cycles. It is important to note here that while forming the data-modulated-PRN-code-cycles, the data bits are hold such that they match the locally generated PRN code chip rate. In other words, the data bits are hold such that 1 data bit lasts for 2 ms, as is the case for BeiDou D2 signal.Step 5:An FFT-based correlation is then performed on each of the possible 2*^X^_bit_* data-bit combinations of the incoming BeiDou signal with the locally generated *T_coh_* ms long data-modulated-PRN-code-cycles (*i.e.*, the output of Step 4).Step 6:As the data-bit set length is 2*^X^_bit_*, there will be altogether 2*^X^_bit_* chunks of correlation matrices with all possible code delay and carrier Doppler combinations for a specific BeiDou GEO satellite. The winning index is the one which has the maximum correlation peak, and therefore, it can then be used for detecting the presence of the satellite, along with the estimation of the carrier Doppler and the code phase via a pre-detection threshold computed against a certain probability of false alarm.

**Figure 4.**
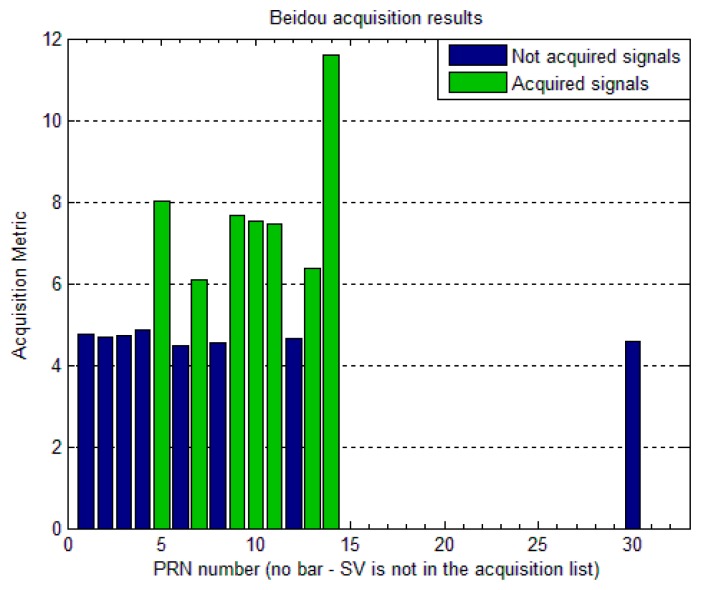
Acquisition metric for BeiDou GEO, IGSO and MEO satellites.

The resultant acquisition metric after utilizing the above two acquisition strategies for BeiDou IGSO/MEO and GEO satellites is shown in Figure 4. BeiDou PRNs 05 (GEO); 07, 09, 10 (IGSO); 11, 13, 14 (MEO) were acquired successfully, which were in view with 10^0^ or more elevation angle at the time of data collection in Finnish Geodetic Institute, Finland.

### 2.2. BeiDou Signal Tracking

The acquisition approaches mentioned earlier in Section 2.1 provide the initial estimates of the carrier Doppler and the code offset. After the acquisition, the control will be handed over to the tracking loops to track the variations of carrier phase and code offset due to the line of sight movement between the satellites and the receiver.

#### 2.2.1. Carrier Tracking

The main objective of signal tracking is to wipe off the code and the carrier. The carrier tracking loop synchronizes the carrier frequency and phase with that of the incoming signal. One of the most commonly used frequency discriminator in a conventional GPS Frequency Locked Loop (FLL) is a four-quadrant arctangent discriminator. This discriminator is optimal at high and low C/N_0_ with a wide frequency pull-in range and it offers a linear relationship between the discriminator output and the real frequency error. This discriminator can not only enhance the robustness of signal tracking but also tolerate large acquisition frequency errors coming from the acquisition. But, this four-quadrant arctangent discriminator is sensitive to data bit transition, meaning that two adjacent integration samples should be within the same data bit interval. In case of legacy GPS L1 signal, the data bit transition may occur only after every 20 ms offering a low data bit rate. Due to the low data bit rate, the GPS receiver can meet this condition in most occasions. Therefore, the probability that the data bit transition affects the discriminator is relatively small (less than or equal to 5%), and the four-quadrant arctangent discriminator based FLL can work correctly [19]. However, in case of BeiDou D1 or any modern GNSS signals with an extra tier of modulation on top of data bits, the bit transition usually occurs rather quickly, and therefore, the four-quadrant arctangent discriminator based FLL is not an appropriate choice for BeiDou D1 signal tracking. The same observation is true for BeiDou D2, where the data bit lasts for only 2 ms, with a maximum probability of data bit transition of 50%. Therefore, the BeiDou receiver should choose an FLL discriminator that is insensitive to data bit transition. In view of this particular situation, a two-quadrant arctangent discriminator is implemented in the software receiver, which is first proposed in [8]. The implemented two-quadrant arctangent discriminator is insensitive to data bit transition, but it has reduced tolerance of frequency uncertainty coming from the acquisition stage. It was shown in [8] that the frequency uncertainty tolerance is reduced by half, as compared to the conventional four-quadrant arctangent discriminator. This restriction in frequency uncertainty can be overcome by proper selection of coherent integration time (*i.e.*, frequency bin size) at the acquisition stage. The signal tracking is switched from FLL to a costas Phase Locked Loop (PLL), once the FLL is locked.

#### 2.2.2. Code Tracking

**Figure 5.**
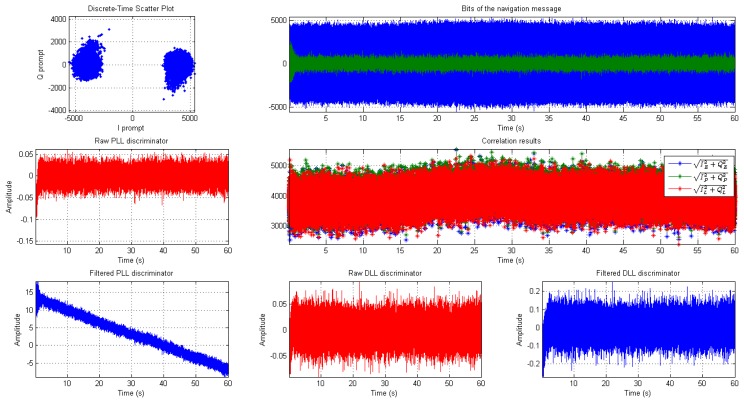
Channel tracking status for PRN 14.

The code tracking loop or Delay Locked Loop (DLL) synchronizes the code phase of the local replica with the incoming signal. A Narrow Correlator (NC) discriminator [20] is implemented in the software receiver as a conventional DLL with early and prompt correlator spacing of 0.1 chips. Figure 5 below shows the tracking status of BeiDou PRN 14 for a 60 s long data.

### 2.3. Bit Boundary Detection

Once the BeiDou receiver keeps tracking the carrier phase and the code offset of the incoming signal, the next phase is to detect the bit boundary and then to wipe off the NH code. The purpose of bit boundary detection is to avoid integration across a data bit-edge which might cause errors in the navigation message detection. Algorithms for the bit boundary detection can be found in [16,21,22]. The Histogram Method, for instance, senses the bit sign changes and keeps a statistic of their position. But this approach will not work with the BeiDou D1 signal due to the presence of NH code. The sign changes in this code within the data bit boundary would in fact be detected as data bit changes affecting the statistics that this method uses for the bit boundary detection. On the other hand, as the data bits are now modulated with the NH code, a simple correlation of the incoming NH code modulated data with the locally generated NH code can then be used to estimate the bit edge. The index with a maximum correlation peak of 20 will be perfectly aligned with the NH code, and it can then be used as the bit boundary index. In case of BeiDou D2 signal, a histogram-based approach is implemented for D2 bit edge detection.

### 2.4. Carrier-to-Noise Density Ratio (C/N_0_) Estimation

The Carrier-to-Noise density ratio (C/N_0_) for the GPS receivers is often calculated based on the Narrow-band Wide-band Power Ratio (NWPR) of the received signal [21]. If this C/N_0_ estimation technique is used, the NH code (in case of D1 navigation signal) must have to be wiped off before the narrowband power is calculated. Otherwise, the narrowband power calculation will be erroneous due to the presence of bit transition within the 20 ms bit boundary. In case of D2 navigation signal, the narrowband power computation may not be accurate enough since the data bit duration for D2 signal is only 2 ms, which is 10 times less than GPS L1 C/A or BeiDou D1 data bit duration. In view of this particular issue, a new C/N_0_ estimation technique based on the Signal-to-Noise Power Ratio (SNPR) is implemented in the FGI-GSRx software receiver. The implementation scheme is unique in the sense that the noise power is computed from the tracking channel itself through a +2 chips distant correlator from the prompt correlator. The properties of the Gold codes (both GPS L1 and BeiDou B1 signals are Gold codes) suggests that any auto-correlation with the same Gold codes outside ±1 chip delay from the prompt correlator should either be zero or very close to zero due to the limiting length of the Gold codes itself. A fair choice of +2 chips early correlation index is preferred here, as this correlation index (+2 chips with respect to prompt correlator) will have no impact from the multipath which usually comes as a delay in the late side of the correlation. The implementation scheme for SNPR-based C/N_0_ estimation technique is mentioned in the following.

Step 1:An estimate of the noise power *μ_N_* is obtained first by correlating the incoming signal with the locally generated PRN (Pseudo Random Noise) code with a correlator which is +2 chips early from the prompt correlation index.Step 2:The signal power *μ_S,N_* is computed from the prompt correlation between the incoming signal and the locally generated PRN code after each code integration period *T_coh_* (*i.e.*, 1 ms for GPS L1 or BeiDou B1).Step 3:Signal-to-Noise Power Ratio (*SNPR*), after each code epoch period, can be written as follows:
(1)${\text{SNPR}}_{i}=\frac{\mu_{S_{i}}}{\mu_{N_{i}}}=\frac{\mu_{S,N_{i}}-\mu_{N_{i}}}{\mu_{N_{i}}}$Step 4:Finally, Carrier-to-Noise-density ratio, C/N_0_ can be derived as:
(2)$${C/N_{0_{i}}|}_{dB-Hz}=\left|10\log_{10}\left(\frac{{\text{SNPR}}_{i}}{T_{\text{coh}}}\right)\right|$$

**Figure 6.**
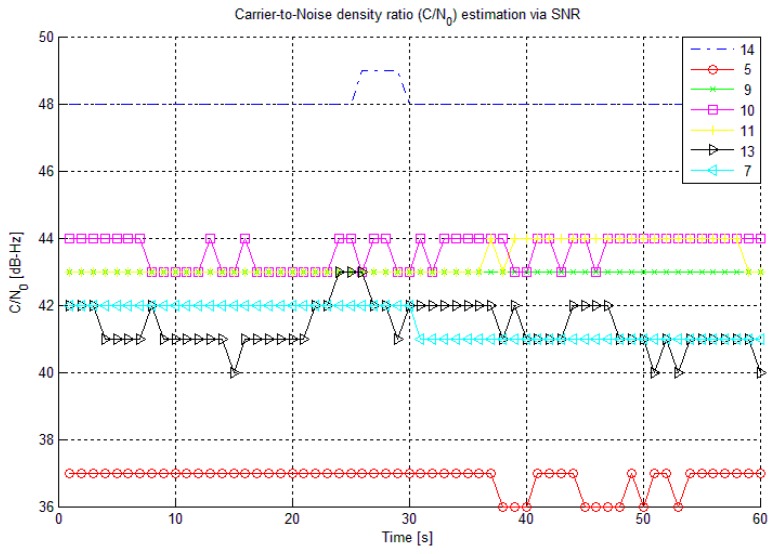
C/N_0_ for the tracked BeiDou satellites.

Figure 6 shows the C/N_0_ of the tracked BeiDou satellites. As shown in the figure, PRN 14 has the highest C/N_0_ (∼48 dB-Hz) and PRN 5 has the lowest C/N_0_ (∼39 dB-Hz) depending on their elevation angles with respect to the receiver at the time of data collection. The performance evaluation of SNPR-based C/N_0_ estimation technique with respect to the traditional NWPR-based technique for BeiDou B1 signal is presented in [23].

### 2.5. Navigation Solution

**Figure 7.**
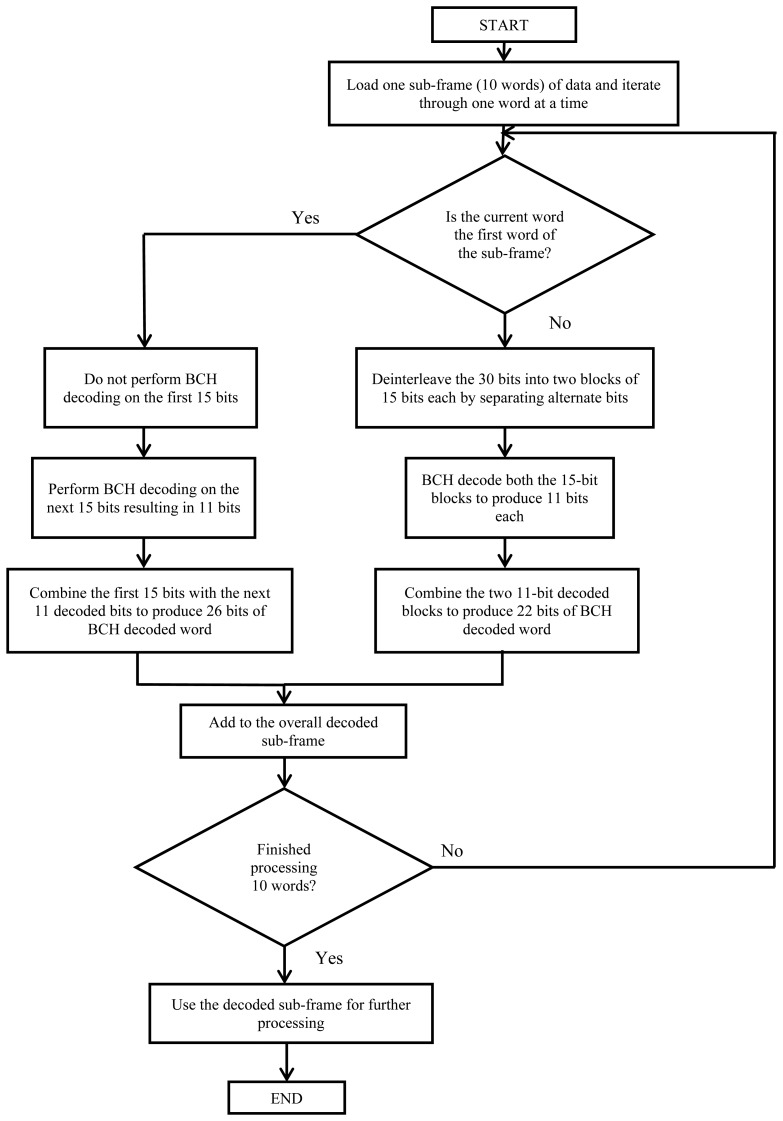
Flowchart illustrating the decoding procedure of BeiDou navigation message.

At the navigation message decoding phase, the first step is to detect the sub-frame preambles on the demodulated data. The BeiDou navigation message has both error correction coding and data interleaving. The error correction is performed by the Bose, Chaudhuri, and Hocquenghem (BCH 15,11,1) codes, which are capable of correcting one-bit error within every block of 15 bits. The decoding process can be illustrated by the flowchart shown in Figure 7. It is important to mention here that the BCH decoding and deinterleaving mechanism is same for both (D1 and D2) navigation messages, and the first word of every sub-frame, the first 15 bits are not BCH encoded. Only the next 11 bits are encoded and hence, this word consists of 26 information bits and four parity bits. Also, data interleaving is not performed in this word. Therefore, the first word of every sub-frame has to be processed differently at the decoding stage within the receiver. After successfully decoding the navigation message, a receiver position can be calculated via a least-square method with at least four visible satellites with decoded ephemerides.

## 3. Live Data Collection and Result Analysis

a dual-frequency front-end from NSL is used to capture the real GNSS data. Among the two front-ends, the maxim 2769B front-end [24] is configured to receive BeiDou B1I signal at 1561.098 MHz, and the maxim 2112 front-end is configured to receive GPS L1 C/A signal at 1575.42 MHz, as mentioned in Table 2. The BeiDou B1I signal spectrum, time-domain plot and bin distribution of the digitized IF samples were already shown in Figure 1 of Section 2.

**Figure 8.**
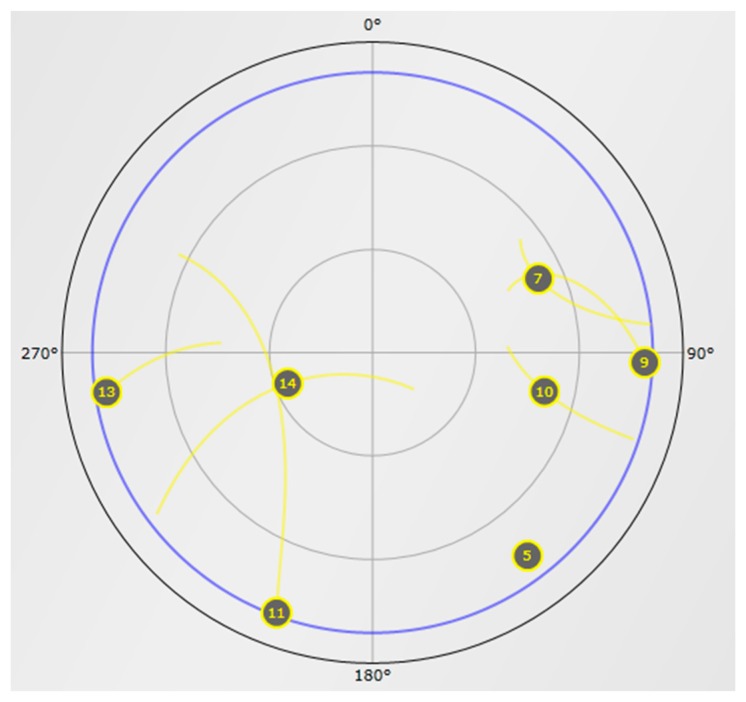
Sky-plot of BeiDou satellite navigation system at UTC time 9:30 AM at Finnish Geodetic Institute with an elevation cut-off angle of 10^0^.

The GNSS data was collected on 31th January at around 9:30 AM UTC time at a static position with a roof antenna in Finnish Geodetic Institute, Kirkkonummi, Finland. The data was collected for about 60 s. The sky-plot for BeiDou constellation at the time of data collection is shown in Figure 8. There are one GEO satellite (PRN 05), three IGSO satellites (PRNs 7, 9, 10), and three MEO (PRNs 11, 13 and 14) satellites available at the moment of data collection. The FGI-GSRx receiver can acquire, track and compute a navigation solution with all the visible BeiDou satellites. The stand-alone positioning results with BDS are presented first followed by GPS-only and multi-GNSS positioning results with BeiDou and GPS.

**Figure 9.**
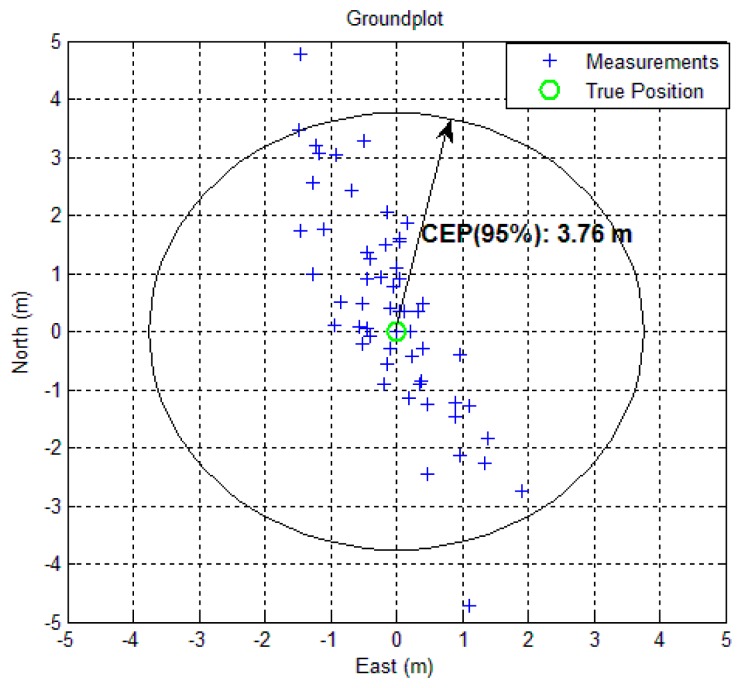
Horizontal error scatter plot.

**Figure 10.**
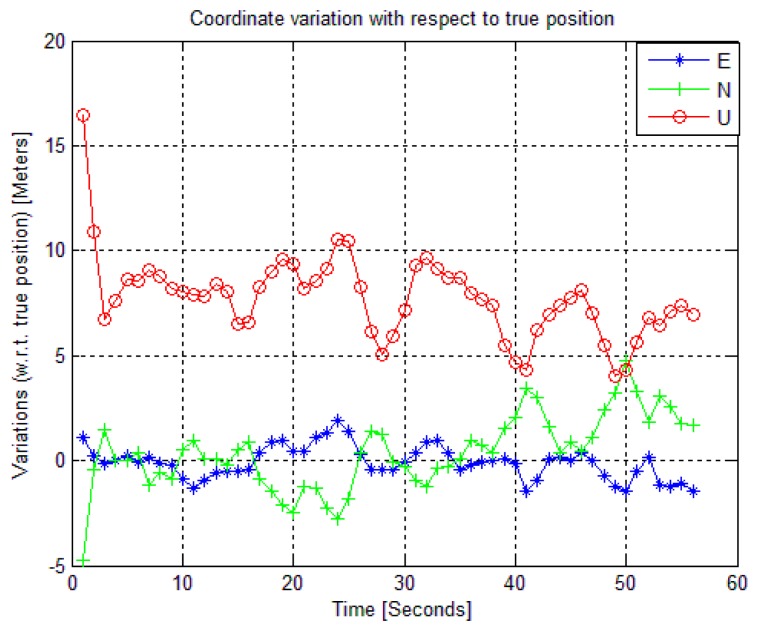
Position error variations with respect to true position in ENU frame.

**Figure 11.**
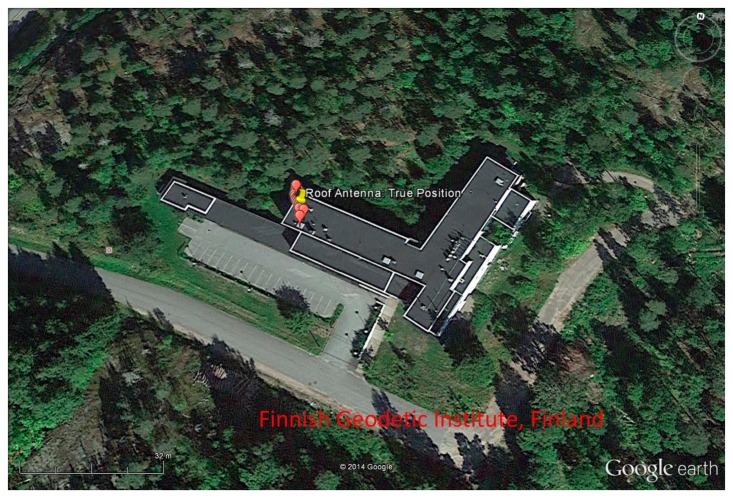
Position fix with BeiDou Satellite Navigation System in Google Earth.

The horizontal error scatter plot and the position error variations in ENU frame are shown in Figures 9 and 10, respectively for BeiDou-only position fix. The 95% Circular Error Probable (CEP) for BeiDou-only horizontal position fix is within 3.76 m. The BeiDou-only position fix is also shown in Google Earth in Figure 11.

The position error statistics for BeiDou, GPS, and multi-GNSS solutions are finally presented in Table 3. The error statistics were computed for a stand-alone code-phase based position solution. The broadcasted Klobuchar ionospheric model parameters are utilized to calculate the ionospheric corrections. The position error statistics were computed with respect to the true known position. The 3D RMS errors for BeiDou and GPS are 8.24 m and 2.80 m, respectively. The error contribution for BeiDou is coming from the vertical component, which is partly due to the fact that four out of seven satellites are low elevated, and hence, the broadcast ionospheric corrections are less accurate with those noisy measurements. Both BeiDou and GPS have comparable horizontal accuracy, whereas GPS outperforms BeiDou in terms of 3D RMS. In case of Multi-GNSS position fix, only the best two BeiDou satellites with the highest elevation angles were considered along with the eight GPS satellites for position computation. The improvement in 3D RMS is really minor with two extra measurements, but the improvement in PDOP is really significant. This eventually means that the multi-GNSS benefits will be more noticeable in harsh GNSS environment than in normal outdoor scenarios with good satellite visibility. In addition to that, the multi-GNSS can certainly offer higher reliability with the addition of new satellites from different constellations.

## 4. Conclusions

The main functionalities of a software-defined BeiDou B1 receiver were presented, while highlighting the similarities and differences of BeiDou B1 signal with the existing GPS L1 C/A signal. A novel acquisition scheme for long coherent integration in the presence of NH code was presented and implemented for acquiring BeiDou IGSO and MEO satellites. Furthermore, a similar phase-preserved acquisition scheme was implemented for BeiDou GEO satellites acquisition. Real GNSS data was collected with a dual frequency front-end, which is then processed with the implemented software-defined GNSS receiver. The positioning results were presented for different GNSS constellations for a static scenario with 60 s long dataset. It was shown that the BeiDou has comparable positioning performance with that of GPS, provided that both the systems have somewhat similar DOP values. A true multi-GNSS positioning results were also computed with GPS and BeiDou systems. The true benefits of multi-GNSS will be more noticeable in harsh GNSS environment with obstructed sky view. The future work includes investigating the performance of a multi-GNSS software-defined receiver in harsh multipath environments (*i.e.*, in urban canyons), as well as developing a Receiver Autonomous Integrity Monitoring (RAIM) system considering the signal quality of individual satellites from different GNSS constellations.

## Figures and Tables

**Table 1.** NSL stereo v2 front-End configuration for BeiDou B1I signal reception.

**Parameter**	**Value**
Intermediate Frequency	6.5 MHz
Front-end Bandwidth	4.2 MHz
Sampling Frequency	26 MHz
Number of Quantization bits	2 bits

**Table 2.** NSL stereo v2 front-end configurations.

**Parameter**	**Max2769B Front-End**	**Max2112 Front-End**
Received signal	BeiDou	GPS
Intermediate Frequency	6.5 MHz	0 MHz
Front-end Bandwidth	4.2 MHz	6.6 MHz
Sampling Frequency	26 MHz	26 MHz
Number of Quantization bits	2 bits	2 bits

**Table 3.** Position error statistics with respect to true position.

	**BeiDou**				**GPS**				**Multi-GNSS**			
	**East**	**North**	**Up**	**3D**	**East**	**North**	**Up**	**3D**	**East**	**North**	**Up**	**3D**
RMS	0.77	1.78	8.0	8.24	1.19	1.77	1.82	2.80	1.0	1.76	1.88	2.77
PDOP	2.05				1.87				1.81			
